# ‘Not Angels but Humans’ An Exploratory Qualitative Study of Female Nurses With Lived Experience of Self‐Harm and Suicidal Behaviours

**DOI:** 10.1111/jan.17013

**Published:** 2025-05-19

**Authors:** Samantha Groves, Keith Hawton, Karen Lascelles

**Affiliations:** ^1^ Oxford Health NHS Foundation Trust Oxford UK; ^2^ Centre for Suicide Research, Department of Psychiatry University of Oxford Oxford UK; ^3^ Senior Lecturer, Mental Health Nursing Oxford Brookes University Oxford United Kingdom

**Keywords:** female, interventions, lived experience, nurses, qualitative study, self‐harm, suicide

## Abstract

**Aims:**

To explore the experiences of qualified nurses who have lived experience of self‐harm (with or without suicidal intent) during nursing training or practice. Specifically, to examine characteristics and contributing factors and ideas for tailored suicide prevention interventions.

**Design:**

Exploratory qualitative study.

**Methods:**

Individual semi‐structured interviews were conducted with eight qualified female nurses who had self‐harmed during nursing training or practice. Participants were recruited from three NHS hospital Trusts. Data were collected between June and September 2023 and analysed using reflexive thematic analysis.

**Results:**

Four themes were generated: (1) ‘I don't think work triggered it, but I don't think it helped’: characteristics and contributors to self‐harm, (2) ‘You're a nurse now you can't talk about that’: nursing culture and barriers to workplace support seeking, (3) ‘Are you a nurse or are you a lived experience practitioner – can you be both?’: navigating a dual identity as a nurse with lived experience and (4) ‘We need the permission that it's ok to put us first’: workplace support and suggestions for suicide prevention.

**Conclusion:**

Participants described their experiences of self‐harm, including citing a range of contributory factors, with occupational issues being particularly salient. Cultural expectations and stigma prevented help‐seeking and unique challenges regarding being both a clinician and an individual who has self‐harmed were described. Reflections and perspectives on workplace and independent mental health support for nurses were shared.

**Implications for the Profession:**

Potential avenues for suicide prevention interventions tailored for the nursing profession may include challenging nursing culture and promoting help‐seeking, peer support opportunities and implementation of education surrounding mental health and well‐being in nursing curricula.

**Reporting Method:**

Reporting complied with the COREQ.

**Patient or Public Contribution:**

The topic guide and participant information sheet were developed in consultation with a group of qualified and student nurses with lived experience of suicidal thoughts and behaviours.

## 1 Introduction

International studies have highlighted nursing as an occupation associated with an increased risk of suicide (Groves et al. 2023), including data from England showing a heightened risk in female nurses (Windsor‐Shellard and Gunnell 2019). Preliminary evidence from Sweden has demonstrated an increased risk of suicide in female nursing students compared to students in other disciplines, with an increased risk of non‐fatal self‐harm in students of both sexes (Lageborn et al. 2023). Self‐poisoning has been shown to be a common method of suicide or hospital‐presenting self‐harm among nurses, particularly involving medication available by prescription or over the counter (The National Confidential Inquiry into Suicide and Safety in Mental Health 2024; Groves, Lascelles, Bale et al. 2024).

Mixed method studies of nurses who have died by suicide demonstrate that multiple factors are likely ‘entangled’ to contribute to suicidal behaviours (Davidson et al. 2021). Such factors may include problems with mental health, employment (e.g., job‐loss, bullying), interpersonal relationships, substance use and physical health. Similar problems have also been demonstrated among nurses and midwives who presented to a hospital in England following self‐harm over an 11‐year period (Groves, Lascelles, Bale et al. 2024). A study of suicide deaths among nurses in the United States during the COVID‐19 pandemic found additional pandemic‐related issues were cited as contributory, including increased workload and associated potential for mistakes, trauma of working within a pandemic, potential infection transmission as well as political and financial factors (James et al. 2023).

The above studies provide some insights into the complexity of issues which may be related to self‐harm and suicidal behaviours among nurses. However, two systematic reviews conducted by the research team which examined suicide and self‐harm among nurses and student nurses found that the perspectives of nurses with personal experience of suicidal behaviours are largely absent from the literature (Groves et al. 2023; Groves, Lascelles, and Hawton 2024). Indeed, a more recent investigation of suicide among nurses in England has called for a detailed qualitative examination of the experiences of nurses related to suicidal behaviours and job‐specific stressors (The National Confidential Inquiry into Suicide and Safety in Mental Health 2024). Hearing the perspectives of nurses who have lived through self‐harm, and/or suicidal thoughts and behaviours, will increase our understanding of potential contributory and protective factors and possible drivers of method choice, which will be valuable to tailor prevention and support initiatives to meet the needs of nursing professionals.

## 2 The Study

### 2.1 Aims

For this study, we aimed to develop an understanding of the experiences and views of female qualified and registered nurses who have self‐harmed during their nursing training or practice.

## 3 Methods

### 3.1 Design

This study involved qualitative semi‐structured interviews. A topic guide (Data S1) was developed based on findings of systematic reviews related to suicide and self‐harm among nurses and student nurses (Groves et al. 2023; Groves, Lascelles, and Hawton 2024). In addition, three qualified and student nurses with lived experience of suicidal thoughts or self‐harm reviewed the proposed topic guide and participant information sheet, assessing wording and identifying additional topics of interest to be explored within interviews. Prior to recruitment, the interview was piloted with a psychiatric nurse with extensive experience of conducting psychosocial assessments with nurses and other individuals following episodes of self‐harm. During the interviews, the researcher (SG) used the topic guide flexibly, whereby participants were encouraged to discuss relevant issues not covered by interview questions. The interviews were broadly focused on exploring characteristics of self‐harm (e.g., method and intent), factors participants thought had been contributory, strengths and challenges of being a nurse with lived experience of self‐harm, and ideas for how suicide prevention interventions and support could be developed or improved for nurses.

### 3.2 Theoretical Framework

This study adopted a critical realist perspective assuming a realist ontology alongside a constructivist epistemology. This approach assumes that experiences can be shared; however, such experiences are inevitably influenced by individual experiences (Koopmans and Schiller 2022). Critical realism has been described as well suited for ‘generating explanatory accounts of complex, multi‐layered and “co‐determined” phenomena’ (Bhaskar and Danermark 2006; Karadzhov 2021) and has been previously used to explore women's experiences of self‐harm (e.g., Curtis 2018). This philosophical position shaped the study design, data collection and analyses.

### 3.3 Study Setting and Recruitment

Participants were recruited from three National Health Service (NHS) hospital Trusts in England using email invitations and study advertisements within each Trust's intranet or newsletters. Invitations contained a central study email address for individuals to indicate interest in participation or to ask questions prior to recruitment. Only the data‐collecting researcher (SG) had access to the personal details of potential participants (to protect participants' privacy).

### 3.4 Inclusion Criteria

Female qualified and registered nurses were eligible to participate if they had self‐harmed during their nursing training or while practising as a registered nurse. For the purposes of this study, self‐harm was defined as non‐fatal intentional self‐poisoning, self‐injury or a combination of methods, irrespective of intentionality (Hawton et al. 2003; National Institute for Health and Care Excellence 2020).

### 3.5 Data Collection

Data were collected using semi‐structured interviews between June and September 2023. Prior to interviews, prospective participants were provided with a participant information sheet and consent form and were invited to ask any questions. A summary sheet containing topics to be covered during the interview was also offered to help participants prepare for the interview and highlight any areas which they did not feel comfortable discussing. Prospective participants who contacted the research team after recruitment had finished, or who were currently not eligible (e.g., student nurses), were offered an information sheet containing the contact details of national and regional suicide prevention and support programmes. They were also asked whether they would like to be contacted regarding future related research. No participants were previously known to the researcher who conducted the interviews (SG).

Participants were offered three interview formats: face‐to‐face (at a private office at the researcher's workplace), by telephone, or online. Regardless of interview format, only audio data were collected. Following each interview, basic demographic data were recorded (age, specialism, years of experience).

An optional simple mood rating scale was used before and after each interview to monitor mood changes associated with interview participation. The scale ranged from 0 being the worst possible mood and 10 being the best possible mood. Similar scales have been previously used in qualitative studies examining experiences related to suicide and self‐harm (Biddle et al. 2013). Following each interview, participants were asked whether they wanted to discuss their experience of the interview. All participants were offered a check‐in phone call the following day and provided with a debrief sheet containing information about local and national support sources related to suicide prevention. Participants were also offered the opportunity for a short follow‐up interview in case of additional reflections identified by the participant. However, no participants chose to participate in such interviews.

After each interview, reflexive field notes were compiled, and the researcher had the opportunity to discuss their reflections with other members of the research team (KL nurse consultant; KH, professor of psychiatry). Recruitment of participants was conducted until the research team agreed that the data collected were sufficient to address the aims of this exploratory study. Traditional assumptions of data saturation were not adhered to in this study. Suicide is a complex phenomenon, where every person who experiences suicidal thoughts and behaviours has a unique experience. It would therefore be impossible to expect to identify all possible themes within a single study, especially in the context of a small‐scale exploratory study (O'Reilly and Parker 2012).

### 3.6 Data Analysis

Audio recordings were transcribed verbatim by one researcher (SG). Potentially identifiable information was anonymised, and audio recordings were deleted following accuracy checking of transcripts. Data were then transferred into NVivo R1 and analysed using reflexive thematic analysis (Braun and Clarke 2019). Iterative data analysis was conducted by one researcher (SG), with consultation with other team members (KL and KH) as required. The first stage of analysis consisted of data familiarisation using transcription as a familiarisation tool alongside note taking. Inductive and deductive coding was then conducted by reading and re‐reading transcripts line by line. Codes were informed by salient issues in the literature regarding suicide in nurses, the aims of the study, issues emphasised as important by Patient and Public Involvement (PPI) representatives (*N* = 3) and issues inductively identified during the analysis. Codes were grouped to generate initial themes, which were subsequently reviewed and developed during discussion with the wider research team. This included one team member (KL) studying each interview transcript and reviewing these in relation to the proposed themes. Themes were revised until the research team agreed the final structure adequately represented the data set and study aims. The findings below are reported in some cases with numerical values. We have made this decision to encourage readers to not overgeneralise the findings of this small scale study to the wider population of all nurses who have self‐harmed. These values did not inform data analysis.

### 3.7 Ethical Considerations

The study received approval from the Health Research Authority (323110). All individuals provided written informed consent prior to participation.

### 3.8 Rigor and Reflexivity

Interviews were conducted by a female research assistant with postgraduate training in qualitative research methods (SG). This researcher has an undergraduate and master's degree in psychology and does not hold a nursing qualification. We were mindful that having a female interviewer and female participants likely influenced data collection and analysis. For example, there was discussion and attention regarding expectations of women in the workplace.

The researchers engaged in several measures to enhance study rigour as characterised by Lincoln and Guba's (1985) criteria of credibility, transferability, dependability and confirmability. The current study was developed after the research team conducted and published several studies related to suicide among nurses, including two systematic reviews (Groves et al. 2023; Groves, Lascelles, and Hawton 2024). This extensive engagement in the research literature supported the design of the study (e.g., questions developed for the interview guide). Furthermore, to enhance credibility of the data analysis, two researchers (SG and KL, a nurse consultant with expertise in suicide prevention) independently analysed all interview transcripts and discussed their interpretation of the data. This practice was conducted not to promote a ‘correct’ analysis but instead to facilitate awareness of other perspectives, and areas which were salient to each researcher. To aid judgement regarding transferability of results, a description of the characteristics of participants is given in the Results section, balancing level of detail with the need to protect participant anonymity in this sensitive study. Dependability was promoted by following a well‐established method for reflexive thematic analysis, and clearly documenting steps taken during data analysis to arrive at the final thematic structure. Finally, confirmability was supported by frequent discussion of the proposed findings as a research team, with experts in the field, and by comparison of findings with the research literature. Throughout the study the researcher also kept a reflexive diary of experiences and views of each interview during data collection and analysis.

## 4 Findings

### 4.1 Characteristics of Participants

Eight female qualified and registered nurses with lived experience of self‐harm during nursing training or practice participated in the interviews. Seven interviews were conducted online using Microsoft Teams, and one interview was conducted face‐to‐face. The length of the interviews ranged from 43 to 95 min (median 59 min). Nurses had a median age of 41.5 years (range 23–61 years), with a median of 15.0 years of experience since qualification (range 10 months–30 years). Most nurses were trained in mental health nursing (*N* = 5), followed by adult nursing (*N* = 3). One nurse was qualified in learning disability and one in children's nursing. Two nurses were dual trained (one in mental health and children's nursing, the other in mental health and learning disability nursing).

Median mood rating scores decreased slightly from 7.5 at the beginning of the interviews to 7 at the end. Five participant's mood scores did not change, two participant's moods decreased and one participant's score increased. Comments made by participants regarding their interview experience included reflections on the difficulty and sadness of the subject, familiarity discussing experiences, and a feeling of catharsis, including reflection on ‘how far they had come’. No participants withdrew from the study or requested that their data be removed.

### 4.2 Thematic Analysis

Thematic analysis generated four themes: (1) ‘I don't think work triggered it, but I don't think it helped’: characteristics and contributors to self‐harm; (2) ‘You're a nurse now you can't talk about that’: nursing culture and barriers to workplace support seeking; (3) ‘Are you a nurse or are you a lived experience practitioner – can you be both?’: navigating a dual identity as a nurse with lived experience and (4) ‘We need the permission that it's ok to put us first’: workplace support, and suggestions for suicide prevention interventions. Quotes are presented below alongside participant numbers. Further information regarding participants (e.g., age, nursing specialism, pseudonyms) has not been presented next to quotes to help preserve participant anonymity.

#### 4.2.1 Theme 1: ‘I Don't Think Work Triggered It, but I Don't Think It Helped’: Characteristics and Contributors to Self‐Harm

All participants reported one or more episodes of self‐harm during their time as a student or qualified nurse. Self‐poisoning by overdosing with medication was used by five participants, and another participant described a near overdose that was prevented when her family intervened. Four participants described self‐injury such as scratching, pinching or hitting, alongside other behaviours they conceptualised as self‐harm, such as restrictive or binge eating and reckless activities (e.g., dangerous driving). Self‐harm was described by participants as having varied functions, including as a means of emotional regulation or release or as a coping mechanism to facilitate an individual continuing to work. Four nurses reported self‐harming and/or experiencing mental health difficulties before nursing registration.

Episodes of self‐poisoning were usually described by participants as having suicidal or ambivalent intent. For example, one participant described wanting *‘to do something that wasn't a definite thing’*, while also feeling ambivalent about living: *‘I didn't really care whether I woke up’* [Participant 2]. Nurses who reported self‐poisoning described taking medication (sometimes multiple types during a single episode) based on *‘what was accessible’* [Participant 8]. Three nurses had taken psychotropic medication prescribed for mental health difficulties, and two had taken prescription or over‐the‐counter medication for physical health conditions. No participants said they had taken medication from their workplace for self‐poisoning. One nurse described her professional identity as protective, where consideration of taking patient medication made her realise that she needed support:

I remember I was on my way […] to a patient and […] I very nearly took like, took the patient's medication that I had on me. And that was where I thought I can't, that, that's a line that I couldn't, that I shouldn't, I shouldn't have even have thought it while being at work (Participant 1)

When reflecting on specific factors contributing to participants' self‐harm, a wide range of problems were reported. These included interpersonal issues (e.g., abusive relationships, conflict, concerns about their children), bereavement, stress, mental and physical health difficulties, financial problems, negative childhood experiences and occupational or training‐based problems. Three participants suggested that factors related to women's health and hormones (e.g., menopause, post‐partum, hormonal contraception) might have been contributory. A combination of stressors inside and outside of work was usually described by participants as provoking their feelings of being overwhelmed and unable to see a way out. Distress appeared to be compounded when support from management was sought but not given:

I know that you can have a rubbish home life but works ok, or vice versa, but when you've got the two or the two and maybe another, another aspect or facet then there's not let off (Participant 7)

[I] tried to get some support from the managers, and they were just angry at me and bullying me because I had taken so much time off, that I shouldn't have done that, basically so that's the first bit that sparked it (Participant 5)

Specific occupational stressors described by participants included workload and associated pressures (e.g., staff shortages, excessive working hours), difficult transitions (moving from a student to qualified role, or into a management position), work or placement‐based incivility, workplace trauma during COVID‐19, errors at work and bullying. Three participants who experienced bullying described this as being a particularly strong factor contributing to their self‐harm:

[…] I thought that [working with patients who are suicidal] would trigger me, if I was going to be triggered. But it wasn't that way at all. It was more, how I felt at work, how disrespected I felt, how unvalued I felt, how controlled I felt, how blinded I felt, and how I felt like I couldn't do anything right, there was no getting it right. How I felt like I was in danger, I was being struck off or something you know? (Participant 1)

#### 4.2.2 Theme 2: ‘You're a Nurse Now You Can't Talk About That’: Nursing Culture and Barriers to Workplace Support Seeking

All participants discussed cultural expectations and attitudes surrounding the nursing profession and the impact of this on their lived experience. Participants were of the view that nurses are often represented in society, and sometimes organisationally, as *‘angels’* or *‘robots’*—selfless individuals with inherent hardness and resilience, immune to the emotional and physical impact of work. Some participants alluded to resilience taking on a new *‘toxic’* meaning in the workplace, placing burden on individual nurses to cope with extreme pressure and stress without recognising organisational responsibility. Furthermore, participants described feeling that normal emotional reactions to traumatic situations encountered at work were almost disallowed due to the focus on resilience. Excessive working was described as expected, and nurses were viewed as *‘disposable’*. One participant described a sense of an expectation to *‘work yourself to the death otherwise you're not doing it properly’* [Participant 6] and that this was present right from commencing nursing training.

Despite participants noting recent improvements in discussion of mental health generally in the workplace, overall, a perceived reluctance was described among nurses regarding talking about their own mental health, including self‐harm. Participants feared that disclosing personal experience of mental health difficulties or self‐harm could be viewed by colleagues as a sign of weakness, with potential negative personal and professional impacts. For example, damaged reputation, judgements regarding their ability and competence as a nurse, and hampered career progression. One nurse stated she *‘didn't want anybody to think of me any less just because of […] the experiences I've had’* [Participant 3].

I think it's probably an assumption, an internal assumption, that when you disclose information to a colleague or to a manager or to someone else, they will know things about you, they will maybe use that against you, they might judge you later on, they might see you in a different way, they might not respect you (Participant 4)

Linked to these fears, participants described concerns about confidentiality regarding help‐seeking. These concerns frequently related to colleagues or managers inappropriately sharing personal information and the fear of colleagues having easy access to electronic records, despite regulations against such inappropriate use. In some cases, these concerns prevented help‐seeking altogether or drove participants to take actions such as seeking care in different geographical locations:

[my manager] doesn't really stick to confidentiality a lot when it comes to staff members. So, I wouldn't want [them] telling other people (Participant 3)

I didn't want to tell people because then they could look up my medical records because obviously everything is online. And I know information governance is not that crash hot and you know you can access things and there is no sort of double check and so that's why I didn't want to tell everyone (Participant 8)

Some participants feared judgement or negative career effects. Two participants described colleagues being moved around the organisation, from team to team, due to mental health issues. Furthermore, all participants described having experienced negative or unhelpful interactions when attempting to access support or discuss their mental health within the workplace. These interactions were with a range of individuals, such as management, colleagues, occupational health services, or human resources. At times, these experiences prevented further disclosure and compounded distress:

She basically questioned, […] ‘are you sure this is the right job for you?’. she said ‘I'm not sure if you're the right person to do this job and if you're well enough’, as in ‘will you ever be’, like me as a person ‘I don't think you're the right person to do it’. […] And I was thinking I'm already feeling extremely unwell […] You start going down the route of I'm worthless, I can't do this and yeah that was another really bad experience (Participant 6)

In comparison, positive work cultures, within which participants felt able to engage in empathic and non‐judgemental conversations, were experienced as helpful. They valued a compassionate approach taken by managers, where difficulties were acknowledged, views were heard and validated, and support was provided, including help with problem solving with the aim of preserving well‐being:

[…] actually, I just spoke to my line manager about it and I half expected him to freak out, but he was, he was really validating, he let me speak and yeah it was really validating. And he didn't freak out and he was like ‘ok it's a thought and that's ok it's a thought and you don't want to act on it and you're communicating with me about it’, and that was very very different. I don't think I wouldn't have felt like that would have been acceptable to do in any other workplace (Participant 1)

#### 4.2.3 Theme 3: ‘Are You a Nurse or Are You a Lived Experience Practitioner – Can You Be Both?’: Navigating a Dual Identity as a Nurse With Lived Experience

Throughout the interviews, participants were invited to reflect on how their life and identity as nurses were shaped by their lived experience. Five participants reported that their experience of negative life events, mental health difficulties, or self‐harm (or these occurring among those close to them) were key factors in their decision to become a nurse. This included being able to give back and provide others with the same positive experience of mental healthcare that they had experienced, or to be able to have a *‘fresh start’*. However, participants also discussed potential difficulties associated with this *‘vocational’* motivation for joining nursing; for example, difficulties setting boundaries, and judgements required by nurses with lived experience to assess their *‘ability to do their role and do it in a way that is professional and safe’* for themselves and patients.

that was the complete reason why I chose to cus I thought about, that I personally when I was in CAMHS [Child and Adolescent Mental Health Services] I had quite a positive experience from it whereas friends and close family that I've had experience CAMHS as well they've all had quite poor experiences. that drove me quite a lot because I was very very thankful for the services that I received so I wanted to be able to give people the same opportunity that I had. (Participant 3)

[when reflecting on the healthcare profession in group counselling] we were both sort of saying because we had really poor childhoods and sort of neglected childhoods you sort of not sort of crave but [you] want to be in an environment where you're sort of wanted, needed. And sort of nursing provides that. But there's, it's only sort of really experience where you learn to put in the boundaries. […] I think there is an element of certain personality types coming in to nursing absolutely. And then your experience in nursing as time goes on is going to trigger so much absolutely trigger so much (Participant 8)

Participants described the value that their lived experience had brought to their practice as a nurse. Experience enhanced empathy and understanding of patients and their carers, particularly for nurses who worked in a mental health field. Additionally, participants reported their experience informing decision‐making regarding what may or may not be effective when helping someone who is distressed, and also gave some participants personal pride in their own recovery journey:

I think it's quite, for me it's been quite empowering being like I've gone from so having really poor mental health, not, yeah not having any like light at the other side of the tunnel sort of thing. to be able to then like know that I can help other people do that, and just yeah, I think it's massively like changed the way that I can speak to people. I can actually just sit there and understand what they're going through, and but also know what sort of like cheesy lines not to say (Participant 3)

Some participants also described the development of professional knowledge and integrity as a nurse as protective for their own mental health. For example, one nurse said that exposure to self‐harm and its effects within her role prevented her from escalating from self‐harm to a suicide attempt, and aided in her recognition that she needed to access support:

One thing that I would say is that being exposed to self‐harm and suicide patients during my practice, somehow protected me from going into a suicide attempt because I could recognise the escalation pattern that I was taking was something that I had seen patients done before. And I had seen the effects and the long‐term effects of what a suicide attempt can bring to your life that actually protected me from going farther. That's what actually made me call for help (Participant 4)

Alongside the inherent value of lived experience, participants also reflected on difficulties experienced in navigating their identity of being both a nurse and a person who has self‐harmed. Some nurses recalled questioning their ability to take on a professional role given their own experiences of mental health difficulties. There were mixed opinions regarding the sharing of personal experiences with patients. Participants highlighted the complexity of this dilemma, where nurses currently do not have clear guidance regarding using and sharing their lived or living experience in practice.

It's I think it's very few people that I patients that I shared it to just because also I don't want to be viewed any differently. I don't want them to just attach that with me and be like ‘well she's got that she can't she can't be professional’ sort of thing. But on the few occasions that I have shared with patients it has seemed to, to help them. (Participant 3)

Some participants described being confronted with situations at work which could be *‘triggering’* for themselves. Encountering patients with similar presentations and witnessing stigmatising attitudes and behaviour towards patients who self‐harm, especially those who did so in the context of diagnosed or assumed personality disorder, was a cause of distress and sometimes disappointment for participants. Observing stigmatising attitudes was described as particularly impactful during training and as junior nurses, where some participants internalised these attitudes and felt unable to challenge them due to fear of exposing of their own lived experience. However, as participants' career's progressed, some felt more able to manage these situations, developing strong personal and professional boundaries, and finding ways of effectively challenging stigmatising discourse using, but not sharing, their experiences:

It's also made me aware of things to avoid. Because I will avoid certain areas talking to people because I know that'll spark mine off‐ me off as well […] There have been people that have been referred to me for therapy before that I've had to refuse because they remind too much, their story reminds me too much to trigger anything (Participant 5)

I imagine there would have been times where I could have stood up and said, ‘well actually I have this experience’, but in those moments I challenged it, but it was from the perspective of a patient not I'm, I was the patient (Participant 1)

#### 4.2.4 Theme 4: ‘We Need the Permission That It's ok to Put us First’: Workplace Support and Suggestions for Suicide Prevention

Alongside mental health resources accessed outside the workplace, all participants discussed their experience of workplace mental health initiatives and support, including reflections on how provision could be improved. Workplace mental health interventions experienced by participants included options for time off, protected hours, employee assistance programmes, and wellbeing resources such as mindfulness or yoga. Several described improvements in the provision of workplace mental health or wellbeing resources in recent years. However, initiatives were sometimes described as feeling tokenistic and not accounting for the experience and knowledge of healthcare staff, nor the unique contexts they are working in. This was described by one participant as resulting in ‘*beginner level’* support, which was not experienced as helpful.

I've been doing mental health and therapy for so long I know what they'll say before they open their mouths […] there's a lot that we do in psychiatric nursing in especially in certain areas like [nursing field] that people in normal life don't come across. So there's a lot we experience that is difficult and different that needs to be understood (Participant 5)

When discussing the development of suicide prevention or mental health interventions tailored for nurses, there was consensus that a universal approach would not be effective for all nurses; instead, a variety of support options should be available to fit individual needs. However, one view frequently expressed by participants was the need to have someone within their workplace that they can talk to about their mental health without judgement. The most frequently suggested person was their manager, who could signpost them for further support. However, related to management, a few participants also highlighted a need for further consideration of support for nurses who experience mental health difficulties or self‐harm while in management or senior positions. It was noted that there is *‘less of a pool of peers that you're able to share some of those grievances or those emotions [with]’* [Participant 7], and managers can be forced into prioritising the needs of other staff above their own.

Some participants suggested that individual or group peer‐support, provided by a nurse who knows *‘the ins and outs’* of the stressors that nurses experience would be valuable. In some cases, participants endorsed the use of such support involving a nurse or group of nurses with their own experiences of mental health difficulties or self‐harm. This was described as having the potential to help nurses realise that they are *‘not alone’* and having the potential to increase help‐seeking:

If I had met someone who gone through that themselves, I would have been more likely to have told them. It's maybe like a peer thing you know (Participant 2)

Participants had varied views about whether peer support would be best placed inside or outside of the workplace, as well as whether support should be in individual or group format. Support independent of the workplace was described as affording participants enhanced anonymity, whereas internal support could be tailored to individual circumstances nurses are working within. Similarly, some felt group settings would be preferable due to the opportunity to build a peer network, whereas others felt individual‐based support would best fit their needs.

I think maybe, maybe away from work because you know, you want to talk about your own stuff, you want to get help. But also you do want to keep your profession and you don't want to blur it you know. But just to know there was somewhere I could have gone, if I needed a counsellor, I could have got a counsellor. Because that's all I needed. (Participant 2)

I personally don't want to sit with a group of people because er I end up focusing on their needs and I put my needs secondary. I personally would need a one‐to‐one. Others who like that camaraderie and that peer that peer support would probably like that. Yeah, I think it needs to be bespoke to the individual (Participant 7)

The need for a cultural shift in nursing was discussed by participants. Related to organisational culture, some nurses expressed a need for realistic expectations in what is expected of nurses as well as recognition of the emotional and physical toll of the role. Participants endorsed the importance of supporting nurses and nursing students to set boundaries to protect their mental health and emphasised the importance of these boundaries being respected within organisations. Some participants suggested challenging management for modelling unrealistic or unhealthy standards because this would not support more junior staff in setting boundaries.

you hear the managers say ‘oh I opened up my laptop on a Sunday’ […] I'm [like] no don't tell them that. Because we don't want that. We don't want that (Participant 2)

Several participants suggested that interventions should be implemented from nursing training onwards. Training was described as a key time for initiatives, before individuals become *‘hardened’* to the current culture of the profession. Interventions during training were described as having the potential to support students with the emotional toll of nursing training and preparing them for the nature of the nursing career, including by setting healthy boundaries in the workplace. In addition, it was suggested that suicide prevention interventions could validate the value of lived experience as a nurse, and help to ensure individuals are aware of where support can be accessed within practice:

[…] I think sort of understanding the pressures and self‐protection and self‐awareness would be great at university because it is, a bit shit generally. So, I think it would be a really useful skill to have (Participant 8)

[…] what I wish I'd had is talking about your own stresses, some learning about how to cope with things rather than get on with the job, do this, do this [intravenous medication] IV do all the rest of it. Learn how to cope with the stresses, being taught that would help. (Participant 5)

## 5 Discussion

We have conducted an exploratory qualitative study that has examined the experiences of qualified female nurses who have self‐harmed, with or without suicidal intent, during nursing training or practice. Participants described the characteristics and factors that contributed to self‐harm episodes, alongside barriers to help‐seeking. Reflections were also shared about the experience of being both a nurse and a person who has self‐harmed and potential avenues for tailored support efforts.

Echoing evidence about self‐harm and suicide among nurses in England (The National Confidential Inquiry into Suicide and Safety in Mental Health 2024; Groves, Lascelles, and Hawton 2024), several participants in the current study had self‐harmed by taking overdoses using medications usually available by prescription or over the counter. However, other forms of self‐harm (e.g., scratching), alongside less‐traditional methods (e.g., reckless behaviours) were also reported. These behaviours are less likely to receive medical attention and may go undetected (Geulayov et al. 2018). Therefore, initiatives established in the community (e.g., universities, workplace), alongside medical settings, may increase access to support and interventions.

As found in studies of nurses, pharmacists and physicians dying by suicide in the United States (Davidson et al. 2021; Groner‐Richardson et al. 2023; James et al. 2023), nurses in the present study identified a wide range of interacting factors contributing to their self‐harm. For some, mental health difficulties and self‐harm preceded nursing training. Occupational stressors highlighted by participants in the present study, such as bullying and excessive workload, have also been reported in nurses engaging in a suicide prevention intervention in the United States (Davidson et al. 2020) and in nurses who died by suicide in England and Wales (Hawton et al. 2002). These findings underlie the importance of addressing and acknowledging occupational stressors encountered by nurses when developing suicide prevention interventions.

Interpersonal issues were also common in the present study, alike nurses presenting to a hospital following self‐harm (Groves, Lascelles, Bale et al. 2024). The contribution of women's health issues to self‐harm was also reported in some interviews. This included use of the contraceptive pill and associated mood changes, pregnancy and postpartum mental health and stages of menopause. Notwithstanding the small number of participants in the study, this finding might be pertinent to female nurses given that there is other evidence showing that such phenomena may contribute to suicidal thoughts and behaviours among some women (Charlton et al. 2014; Knight et al. 2022; Nakanishi et al. 2023). Future studies are warranted examining contributors towards suicidal behaviours among nurses related to sex and gender to inform intervention development. For example, the latest NCISH report indicated that social isolation may be an important factor among male nurses who die by suicide (The National Confidential Inquiry into Suicide and Safety in Mental Health 2024).

Anxieties related to help‐seeking or discussion of mental health within the workplace were described by participants in the current study. This appeared to be underpinned by several concerns and experiences also well documented among healthcare staff and first responders, including nurses, doctors, psychologists, paramedics, fire‐fighters and the police. Such issues include judgments related to professional competence, hampered career progression, stigmatisation by colleagues, shame and concerns surrounding confidentiality (Joyce et al. 2012; Waugh et al. 2017; Tay et al. 2018; Jones et al. 2020; Edwards and Kotera 2021; King et al. 2020; Zaman et al. 2022). The availability of support independent of the workplace, alongside enhanced protection of staff electronic health records, may facilitate help‐seeking by nurses.

Supportive and open workplace cultures have also been shown to be an important facilitator of help‐seeking for healthcare workers (Zaman et al. 2022). Implementation of education by nurses with lived experience of mental health difficulty or suicidality within nursing training may help tackle stigmatising views related to mental health difficulties and promote help‐seeking. In a study of American medical students, 91% endorsed the view that ‘knowing physicians further along in their careers who struggled with mental health issues, got treatment, and were now doing well would make them more likely to access care if they needed it’ (Martin et al. 2020).

In planning mental health support options, there should be consideration of the needs of staff at different career stages; for example, student, junior and senior positions, where stressors and support needs are likely to differ. Peer‐based support was frequently endorsed as a potential intervention for nurses at all stages of their career. General peer support interventions for staff are already present in some clinical settings, for example, peer support groups for psychiatrists following patient suicide or homicide (Tamworth et al. 2022), and peer support for healthcare workers during the COVID‐19 pandemic (e.g., Bridson et al. 2021). However, it is important to note that further work is required to examine the effectiveness of such interventions for suicide prevention (Schlichthorst et al. 2020). If workplace and independent programmes are to be led by nurses with lived experience, it is vital that facilitators are provided with appropriate training, guidance and support. This is important for supporting the well‐being and sustainability of the lived‐experience workforce (e.g., Hawgood et al. 2023).

Participants in this study reflected on holding ‘hybrid’ or ‘dual’ identities as a nurse and a person who has self‐harmed. The value brought to the profession by those with lived or living experience was described, including enhanced empathy and understanding of the needs for patients and carers. Furthermore, experiences promoted nurses to challenge problematic culture in the workplace and recognise changes in their own and colleague's well‐being. However, participants also described the challenges of holding these identities, which have been discussed in qualitative and reflective articles. Such challenges include dilemmas surrounding disclosure, and experiencing stigmatising views from colleagues or patients, alongside internalised stigma (Ramluggun et al. 2018; Butler 2022; Fisher 2023). Further consideration needs to be given to the growing lived experience workforce in nursing, with initial evidence demonstrating possible prior vulnerability related to suicide and self‐harm among individuals entering nursing training (Lageborn et al. 2023). Training and guidance for managers (and university staff) should be developed to promote compassionate and constructive conversations with colleagues or students disclosing lived or living experience. This may promote positive outcomes related to further help‐seeking, and discussions about psychological safety. A recent educational programme has been evaluated in California to support nurse leaders in the recognition and support of nursing staff who may be suicidal. The programme includes education and role‐playing based on suicide‐related risk factors, examples of behaviours that may suggest someone is suicidal, empathic listening techniques, motivational interviewing, and suggestions of resources for nurse leaders to signpost nurses to. The programme led to improvements among nurse leaders related to knowledge and self‐efficacy surrounding suicide prevention (James et al. 2025). To guide development of such initiatives in England, research examining managers' and university staff members' experiences of supporting nurses or student nurses with mental health distress is warranted.

### 5.1 Strengths and Limitations

The findings of this study provide a rich account of the experiences of a sample of nurses with lived experience of self‐harm. From this, we have identified potential avenues for further research and suicide prevention interventions. It is, however, important to note that these accounts are from a small sample of female nurses from a relatively affluent area, who have self‐harmed specifically during nursing training or practice, and who are comfortable talking about mental health issues, based on their area of expertise and their own experience. In accordance with the critical realist perspective of this project, we emphasise that we cannot generalise our findings to all nurses with lived experience of self‐harm. We have therefore included some numerical values throughout to underline the small numbers of those involved.

Job loss has been linked as an important workplace contributory factor for suicide among nurses (Davidson et al. 2021). Due to our recruitment through NHS Trusts, it was not possible for us to explore this as a potential contributory factor. Use of a theoretical framework such as the integrated motivational–volitional model of suicidal behaviour (O'Connor and Kirtley 2018) may help us understand the transition from suicidal thoughts to behaviours among nursing professionals, and to facilitate identification of key time points for targeted interventions. The findings of this study, in combination with previous work by the research team, are currently being used to inform a doctoral research programme (SG is a doctoral student), including further qualitative inquiry. The results of this programme will be used to inform and provide recommendations for the development of suicide prevention interventions.

## 6 Conclusions

This study has provided a detailed account of the experiences of a group of qualified nurses in England who have experienced self‐harm during nursing training or practice. Suicide prevention interventions should be developed both within, and independent of the workplace, and should account for the anxiety nurses with personal experience of mental health difficulties or self‐harm encounter related to stigmatisation. Further exploration of the views of nurses and student nurses with lived experience of self‐harm or suicidal ideation will support the development of suicide prevention interventions tailored to this occupational group's needs.

## Conflicts of Interest

The authors declare no conflicts of interest.

## Supporting information


Data S1.

Data S2.

## Data Availability

The authors have nothing to report. If you are interested in the ongoing programme of work, please contact the team at nurse-study@psych.ox.ac.uk.

## References

[jan17013-bib-0001] Bhaskar, R. , and B. Danermark . 2006. “Metatheory, Interdisciplinarity and Disability Research: A Critical Realist Perspective.” Scandinavian Journal of Disability Research 8, no. 4: 278–297. 10.1080/15017410600914329.

[jan17013-bib-0002] Biddle, L. , J. Cooper , A. Owen‐Smith , et al. 2013. “Qualitative Interviewing With Vulnerable Populations: Individuals' Experiences of Participating in Suicide and Self‐Harm Based Research.” Journal of Affective Disorders 145, no. 3: 356–362. 10.1016/j.jad.2012.08.024. 23021191

[jan17013-bib-0003] Braun, V. , and V. Clarke . 2019. “Reflecting on Reflexive Thematic Analysis.” Qualitative Research in Sport, Exercise and Health 11, no. 4: 589–597. 10.1080/2159676X.2019.1628806.

[jan17013-bib-0004] Bridson, T. L. , K. Jenkins , K. G. Allen , and B. M. McDermott . 2021. “PPE for Your Mind: A Peer Support Initiative for Health Care Workers.” Medical Journal of Australia 214, no. 1: 8–11. 10.5694/mja2.50886. 33294997

[jan17013-bib-0005] Butler, R. M. 2022. “Serious Mental Illness in Healthcare and Academia: A Lived Experience.” Journal of Psychiatric and Mental Health Nursing 29, no. 5: 624–629. 10.1111/jpm.12862. 35876216

[jan17013-bib-0006] Charlton, B. M. , J. W. Rich‐Edwards , G. A. Colditz , et al. 2014. “Oral Contraceptive Use and Mortality After 36 Years of Follow‐Up in the Nurses' Health Study: Prospective Cohort Study.” BMJ 349: g6356. 10.1136/bmj.g6356. 25361731 PMC4216099

[jan17013-bib-0007] Curtis, C. 2018. “Female Deliberate Self‐Harm: The Women's Perspectives.” Women's Studies 47, no. 8: 845–867. 10.1080/00497878.2018.1524762.

[jan17013-bib-0008] Davidson, J. E. , R. Accardi , C. Sanchez , S. Zisook , and L. A. Hoffman . 2020. “Sustainability and Outcomes of a Suicide Prevention Program for Nurses.” Worldviews on Evidence‐Based Nursing 17, no. 1: 24–31. 10.1111/wvn.12418. 32017435

[jan17013-bib-0009] Davidson, J. E. , G. Ye , M. C. Parra , et al. 2021. “Job‐Related Problems Prior to Nurse Suicide, 2003‐2017: A Mixed Methods Analysis Using Natural Language Processing and Thematic Analysis.” Journal of Nursing Regulation 12, no. 1: 28–39. 10.1016/S2155-8256(21)00017-X.

[jan17013-bib-0010] Edwards, A. M. , and Y. Kotera . 2021. “Mental Health in the UK Police Force: A Qualitative Investigation Into the Stigma With Mental Illness.” International Journal of Mental Health and Addiction 19, no. 4: 1116–1134. 10.1007/s11469-019-00214-x.

[jan17013-bib-0011] Fisher, J. 2023. “Who Am I? The Identity Crisis of Mental Health Professionals Living With Mental Illness.” Journal of Psychiatric and Mental Health Nursing 30: 880–884. 10.1111/jpm.12930. 37668545

[jan17013-bib-0012] Geulayov, G. , D. Casey , K. C. McDonald , et al. 2018. “Incidence of Suicide, Hospital‐Presenting Non‐Fatal Self‐Harm, and Community‐Occurring Non‐Fatal Self‐Harm in Adolescents in England (The Iceberg Model of Self‐Harm): A Retrospective Study.” Lancet Psychiatry 5, no. 2: 167–174. 10.1016/S2215-0366(17)30478-9. 29246453

[jan17013-bib-0013] Groner‐Richardson, M. A. , S. A. Cotton , S. Ali , et al. 2023. “Reflexive Thematic Analysis of Job‐Related Problems Associated With Pharmacist Suicide, 2003–2019.” Research in Social and Administrative Pharmacy 19, no. 5: 728–737. 10.1016/j.sapharm.2023.02.001. 36781370

[jan17013-bib-0014] Groves, S. , K. Lascelles , L. Bale , F. Brand , D. Casey , and K. Hawton . 2024. “Self‐Harm by Nurses and Midwives—A Study of Hospital Presentations.” Crisis: The Journal of Crisis Intervention and Suicide Prevention 45, no. 2: 128–135. 10.1027/0227-5910/a000936. PMC10985583 38234244

[jan17013-bib-0015] Groves, S. , K. Lascelles , and K. Hawton . 2023. “Suicide, Self‐Harm, and Suicide Ideation in Nurses and Midwives: A Systematic Review of Prevalence, Contributory Factors, and Interventions.” Journal of Affective Disorders 331: 393–404. 10.1016/j.jad.2023.03.027. 36933670

[jan17013-bib-0016] Groves, S. , K. Lascelles , and K. Hawton . 2024. “Suicidal Thoughts and Behaviours Among Student Nurses and Midwives: A Systematic Review.” Journal of Advanced Nursing 80, no. 6: 2202–2213. 10.1111/jan.15982. 38010816

[jan17013-bib-0017] Hawgood, J. , J. Rimkeviciene , M. Gibson , et al. 2023. “Informing and Sustaining Participation of Lived Experience in the Suicide Prevention Workforce.” International Journal of Environmental Research and Public Health 20, no. 4: 3092. 10.3390/ijerph20043092. 36833786 PMC9963089

[jan17013-bib-0018] Hawton, K. , L. Harriss , S. Hall , S. Simkin , E. Bale , and A. Bond . 2003. “Deliberate Self‐Harm in Oxford, 1990–2000: A Time of Change in Patient Characteristics.” Psychological Medicine 33, no. 6: 987–995. 10.1017/S0033291703007943. 12946083

[jan17013-bib-0019] Hawton, K. , S. Simkin , J. Rue , et al. 2002. “Suicide in Female Nurses in England and Wales.” Psychological Medicine 32, no. 2: 239–250. 10.1017/S0033291701005165. 11866319

[jan17013-bib-0020] James, K. E. , S. Agarwal , K. L. Armenion , et al. 2023. “A Deductive Thematic Analysis of Nurses With Job‐Related Problems Who Completed Suicide During the Early COVID‐19 Pandemic: A Preliminary Report.” Worldviews on Evidence‐Based Nursing 20, no. 2: 96–106. 10.1111/wvn.12640. 36991524

[jan17013-bib-0021] James, K. E. , J. Rogers , R. Accardi , G. Aryal , P. Ludwig‐Beymer , and J. E. Davidson . 2025. “Supporting Nurse Leaders to Recognize and Intervene in Team Members' Suicidality.” Journal of Nursing Scholarship. 10.1111/jnu.70006. PMC12241765 40116005

[jan17013-bib-0022] Jones, S. , K. Agud , and J. McSweeney . 2020. “Barriers and Facilitators to Seeking Mental Health Care Among First Responders: “Removing the Darkness”.” Journal of the American Psychiatric Nurses Association 26, no. 1: 43–54. 10.1177/1078390319871997. 31509058

[jan17013-bib-0023] Joyce, T. , I. Higgins , P. Magin , et al. 2012. “The Experiences of Nurses With Mental Health Problems: Colleagues' Perspectives.” Archives of Psychiatric Nursing 26, no. 4: 324–332. 10.1016/j.apnu.2011.12.003. 22835752

[jan17013-bib-0024] Karadzhov, D. 2021. “Explaining Mental Health Recovery in the Context of Structural Disadvantage: The Unrealised Potential of Critical Realism.” Social Theory and Health 19: 172–185. 10.1057/s41285-019-00122-z.

[jan17013-bib-0025] King, A. J. , L. M. Brophy , T. L. Fortune , and L. Byrne . 2020. “Factors Affecting Mental Health Professionals' Sharing of Their Lived Experience in the Workplace: A Scoping Review.” Psychiatric Services 71, no. 10: 1047–1064. 10.1176/appi.ps.201900606. 32878543

[jan17013-bib-0026] Knight, M. , K. Bunch , R. Patel , et al. 2022. Saving Lives, Improving Mothers' Care Core Report ‐ Lessons Learned to Inform Maternity Care From the UK and Ireland Confidential Enquiries Into Maternal Deaths and Morbidity 2018–20. National Perinatal Epidemiology Unit, University of Oxford. https://www.npeu.ox.ac.uk/assets/downloads/mbrrace‐uk/reports/maternal‐report‐2022/MBRRACE‐UK_Maternal_CORE_Report_2022_v10.pdf.

[jan17013-bib-0027] Koopmans, E. , and D. C. Schiller . 2022. “Understanding Causation in Healthcare: An Introduction to Critical Realism.” Qualitative Health Research 32, no. 8–9: 1207–1214. 10.1177/10497323221105737. 35649292 PMC9350449

[jan17013-bib-0028] Lageborn, C. T. , J. Bjureberg , J. Song , et al. 2023. “Risk of Suicide and Self‐Harm in University Students Entering Different University Programs–a National Register‐Based Cohort Study in Sweden.” Social Psychiatry and Psychiatric Epidemiology 58, no. 8: 1139–1149. 10.1007/s00127-023-02484-2. 37149517 PMC10366015

[jan17013-bib-0029] Lincoln, Y. S. , and E. G. Guba . 1985. Naturalistic Inquiry. Vol. 75. Sage Publications.

[jan17013-bib-0030] Martin, A. , J. Chilton , D. Gothelf , and D. Amsalem . 2020. “Physician Self‐Disclosure of Lived Experience Improves Mental Health Attitudes Among Medical Students: A Randomized Study.” Journal of Medical Education and Curricular Development 7: 2382120519889352. 10.1177/2382120519889352. 32363235 PMC7180952

[jan17013-bib-0031] Nakanishi, M. , K. Endo , S. Yamasaki , et al. 2023. “Association Between Menopause and Suicidal Ideation in Mothers of Adolescents: A Longitudinal Study Using Data From a Population‐Based Cohort.” Journal of Affective Disorders 340: 529–534. 10.1016/j.jad.2023.08.055. 37573891

[jan17013-bib-0032] National Institute for Health and Care Excellence . 2020. “Self‐Harm: What Is It?” https://cks.nice.org.uk/topics/self‐harm/background‐information/definition/.

[jan17013-bib-0033] O'Connor, R. C. , and O. J. Kirtley . 2018. “The Integrated Motivational–Volitional Model of Suicidal Behaviour.” Philosophical Transactions of the Royal Society B: Biological Sciences 373, no. 1754: 20170268. 10.1098/rstb.2017.0268. PMC6053985 30012735

[jan17013-bib-0034] O'Reilly, M. , and N. Parker . 2012. “‘Unsatisfactory Saturation’: A Critical Exploration of the Notion of Saturated Sample Sizes in Qualitative Research.” Qualitative Research 13, no. 2: 190–197. 10.1177/1468794112446106.

[jan17013-bib-0035] Ramluggun, P. , M. Lacy , M. Cadle , and M. Anjoyeb . 2018. “Managing the Demands of the Preregistration Mental Health Nursing Programme: The Views of Students With Mental Health Conditions.” International Journal of Mental Health Nursing 27, no. 6: 1793–1804. 10.1111/inm.12486. 29847011

[jan17013-bib-0036] Schlichthorst, M. , I. Ozols , L. Reifels , and A. Morgan . 2020. “Lived Experience Peer Support Programs for Suicide Prevention: A Systematic Scoping Review.” International Journal of Mental Health Systems 14: 65. 10.1186/s13033-020-00396-1. 32817757 PMC7425132

[jan17013-bib-0037] Tamworth, M. , H. Killaspy , J. Billings , and R. Gibbons . 2022. “Psychiatrists' Experience of a Peer Support Group for Reflecting on Patient Suicide and Homicide: A Qualitative Study.” International Journal of Environmental Research and Public Health 19, no. 21: 14507. 10.3390/ijerph192114507. 36361387 PMC9654625

[jan17013-bib-0038] Tay, S. , K. Alcock , and K. Scior . 2018. “Mental Health Problems Among Clinical Psychologists: Stigma and Its Impact on Disclosure and Help‐Seeking.” Journal of Clinical Psychology 74, no. 9: 1545–1555. 10.1002/jclp.22614. 29573359

[jan17013-bib-0039] The National Confidential Inquiry into Suicide and Safety in Mental Health (NCISH) . 2024. Suicide by Nurses; Update Report (2011–2022). University of Manchester. https://documents.manchester.ac.uk/display.aspx?DocID=74946.

[jan17013-bib-0040] Waugh, W. , C. Lethem , S. Sherring , and C. Henderson . 2017. “Exploring Experiences of and Attitudes Towards Mental Illness and Disclosure Amongst Health Care Professionals: A Qualitative Study.” Journal of Mental Health 26, no. 5: 457–463. 10.1080/09638237.2017.1322184. 28488890

[jan17013-bib-0041] Windsor‐Shellard, B. , and D. Gunnell . 2019. “Occupation‐Specific Suicide Risk in England: 2011–2015.” British Journal of Psychiatry 215, no. 4: 594–599. 10.1192/bjp.2019.69. 30929655

[jan17013-bib-0042] Zaman, N. , K. Mujahid , F. Ahmed , et al. 2022. “What Are the Barriers and Facilitators to Seeking Help for Mental Health in NHS Doctors: A Systematic Review and Qualitative Study.” BMC Psychiatry 22, no. 1: 595. 10.1186/s12888-022-04202-9. 36071392 PMC9450826

