# Supplementary material for: ‘Not Angels but Humans’ An Exploratory Qualitative Study of Female Nurses With Lived Experience of Self‐Harm and Suicidal Behaviours
Source: J Adv Nurs. 2025 May 19;82(1):780–90. doi: 10.1111/jan.17013 (PMC12721943; doi:10.1111/jan.17013)
Supplement: Supplementary file 1 — Data S1. [file JAN-82-780-s002.docx]

**Interview Topic guide**

[Additional prompts were used as appropriate throughout the study, language used informed by that used by participant]

1. Could you tell me a bit about yourself?
2. I was wondering what do you think about the mental health of nurses?
3. If it is ok, we will now move on to your own experiences. Please remember you can choose not to answer any of these questions and can pause and stop at any time. To start could you tell me about your experience of self-harm/ suicide attempts during nursing training/practice?
4. Do you feel there was any relationship between your work/training and the self-harm episode/ suicide attempt?
5. Did you ever inform anybody from your workplace about self-harm episode/ suicide attempt?
6. How do you think your own experiences as a nurse have been altered because of your experiences?
7. Is there anything you think can be done to help prevent/support nurses at risk of suicide or self-harm?
8. I’ve now reached the end of the set questions. Is there anything I didn’t ask you about that you think is important to tell us about this topic?
